# Inhibitory Effects of *Inonotus obliquus* Polysaccharide on Inflammatory Response in *Toxoplasma gondii-*Infected RAW264.7 Macrophages

**DOI:** 10.1155/2021/2245496

**Published:** 2021-12-29

**Authors:** Kexin Yan, Hongyuan Zhou, Meng Wang, Haitao Li, Rui Sang, Bingjie Ge, Xin Zhao, Chunting Li, Wei Wang, Xuemei Zhang

**Affiliations:** ^1^Agricultural College of Yanbian University, Gongyuan Street, Yanji, Jilin 133002, China; ^2^Institute of Special Wild Economic Animals and Plants, Chinese Academy of Agricultural Sciences, Juye Street, Changchun, Jilin 132109, China; ^3^Engineering Research Center of North-East Cold Region Beef Cattle Science & Technology Innovation, Ministry of Education, Yanbian University, Gongyuan Street, Yanji, Jilin 133002, China

## Abstract

Our previous reports have shown that *Inonotus obliquus* polysaccharide (IOP) has protective effects against *Toxoplasma gondii* (*T. gondii*) infection *in vivo*. The aim of the present research is to explore the *in vitro* anti-inflammatory effects of IOP and its mechanism in RAW264.7 macrophages infected by *T. gondii*. In this study, it is indicated that IOP decreased the excessive secretion of inflammatory cytokines tumor necrosis factor-*α* (TNF-*α*), interferon-*γ* (IFN-*γ*), interleukin-1*β* (IL-1*β*), IL-4, and IL-6 in *T. gondii*-infected RAW264.7 macrophages. IOP effectively suppressed the mRNA expression of these cytokines and chemokines monocyte chemoattractant protein-1 (MCP-1) and macrophage inflammatory protein-1*α* (MIP-1*α*). Moreover, IOP inhibited the phosphorylation of inhibitor kappa B kinase *α*/*β* (IKK*α*/*β*), inhibitor *κ*B*α* (I*κ*B*α*), p65 in nuclear factor-kappa B (NF-*κ*B) signaling pathway and p38, c-Jun N-terminal kinase (JNK), and extracellular signal-regulated kinase 1/2 (ERK1/2) in mitogen-activated protein kinases (MAPKs) signaling pathway. Meantime, IOP prevented NF-*κ*B p65 and c-Jun translocation from the cytoplasm to the nucleus. Further, IOP downregulated the protein expression of toll-like receptor 2 (TLR2) and TLR4 in *T. gondii*-infected RAW264.7 macrophages. The above results suggest that IOP can inhibit the inflammatory response infected with *T. gondii* via regulating TLR2/TLR4-NF-*κ*B/MAPKs pathways and exerting its anti-*T. gondii* role *in vitro*.

## 1. Introduction

*Toxoplasma gondii* (*T. gondii*), as an apicomplexan parasitic organism in warm-blooded animals and humans [1], can infect almost all nucleated cells [2]. Toxoplasmosis caused by *T. gondii* is one of the most globally harmful zoonotic diseases with complex epidemiology and multiple manifestations, which has a severe impact on the healthy development of animal husbandry and livestock products, increases the risk of human infection, and seriously threatens human health and public safety [3]. Ordinarily, *T. gondii* infection is recessive and asymptomatic in immunocompetent hosts [4, 5]. When the host's immune function is low or defective, *T. gondii* proliferates *in vivo* and results in systemic inflammatory responses and multiple organ damage [6–8]. Therefore, the inflammatory response is not only an important link in host resistance to *T. gondii* but also an important cause of host pathological damage.

Toll-like receptors (TLRs) are essential pattern recognition receptors involved in innate immunity and expressed differentially among immune cells [9]. TLRs can recognize the glycerophosphoinositide- (GPI-) anchored protein on the surface of *T. gondii* and then activate the downstream nuclear factor-*κ*appaB (NF-*κ*B) and mitogen-activated protein kinases (MAPKs) signaling pathways, the activation of signaling pathways further stimulates multiple immune cells to produce a large number of inflammatory cytokines and chemokines, leading to the inflammatory response [10–12].

Due to the *T. gondii*'s ability to infect any warm-blood animal and the complexity of the life cycle of *T. gondii*, the ideal vaccine for toxoplasmosis has not been applied in clinical practice [13]. The prevention and treatment of toxoplasmosis depend mainly on sulfonamides (such as sulfadiazine and pyrimethamine) and macrolides (such as azithromycin and spiramycin). However, these synthetic drugs are prone to produce drug resistance and serious adverse reactions [14]. Therefore, there is growing interest in deciphering the role of various immunomodulatory compounds derived from natural products on toxoplasmosis; more effective and safer natural products have been widely concerned [15]. *Inonotus obliquus,* also known as *Chaga,* is a medicinal and edible fungus, which belongs to the Hymenochaetaceae family from Basidiomycetes and grows on white birch trees [16]. *Inonotus obliquus* polysaccharide (IOP) is one of the essential active ingredients of *Inonotus obliquus*. It possesses various pharmacological effects such as anti-inflammation, antioxidation, and immunity enhancement and has potential protective effects against a variety of diseases, including cancer, pancreatitis, diabetes, colitis, hyperlipaemia, and Alzheimer's disease [17, 18]. Our series of *in vivo* studies has also found that IOP possesses protective effects on liver injury caused by *T. gondii* infection [19] and *T. gondii*-infected impaired reproductive function in mice [18, 20]. Previous reports in our laboratory have also shown that IOP can inhibit the growth and reproduction of *T. gondii in vitro* and reduce the parasite load in the mouse spleen [21, 22]. However, the *in vitro* anti-*T. gondii* mechanism of IOP is unclear. To further explore the *in vitro* anti-*T. gondii* effect and the mechanism of IOP, we studied the *in vitro* anti-inflammatory effects of IOP in *T. gondii*-infected RAW264.7 macrophages and the immunomodulatory mechanism on related signaling pathways.

## 2. Materials and Methods

### 2.1. Chemicals and Reagents

Sulfadiazine (SD) and 3-(4,5-dimethylthiazol-2-yl)-2,5-diphenyltetrazolium bromide (MTT) were purchased from Sigma (St. Louis, MO, USA). Antifluorescence quenching solution and DAPI staining solution were purchased from Biyuntian Biotechnology (Shanghai, China). Fetal bovine serum (FBS) and Dulbecco's Modified Eagle's medium (DMEM) were purchased from Gibco (Grand Island, NY, USA). Prime Script TMRT-PCR Kit was purchased from Takara (Kyoto, Japan). IL-1*β*, IL-4, IL-6, TNF-*α*, and IFN-*γ* ELISA kits were purchased from BioLegend (San Diego, CA, USA). Primary antibodies p65, p-p38, and p-I*κ*B*α* were obtained from Cell Signaling Technology (Danvers, MA, USA). Primary antibodies TLR2, TLR4, c-Jun, p38, ERK1/2, JNK, p-I*κ*K*α*/*β*, p-ERK1/2, and p-JNK were purchased from Abcam (Cambridge, UK). Horseradish peroxidase-conjugated secondary antibody was obtained from Santa Cruz (Santa Cruz, CA, USA).

### 2.2. Extraction, Purification, and Composition Analysis of IOP

*Inonotus obliquus* was obtained from Hailanjiang Pharmacy (Yanji, China). The extraction, purification, and composition analysis of IOP were as mentioned in our former descriptions [18–20]. Namely, *Inonotus obliquus* was dissolved and protein was excluded with the savage method, followed by dialysis with running water and distilled water. The crude polysaccharide was centrifuged, lyophilized, and further purified in an anion-exchange DEAE cellulose column. The major fraction was further purified on a Sephadex G-200 gel column, collected, dialyzed, freeze-dried to fine powder, and stored in an airtight container at 4°C. Monosaccharide components of IOP were analyzed using the HPLC method. It was achieved in a Kromasil C18 column, the mobile phase was methanol and 50 mmol/L phosphoric acid buffer, and the flow rate was 1.0 mL/min.

### 2.3. Culture of Cells and *T. gondii*

Mouse RAW264.7 macrophages and Vero cells were from Kebai Biotechnology (Nanjing, China). The virulent RH strain of *T. gondii* was generously donated by the National Research Center for Protozoan Diseases, School of Agriculture and Veterinary Medicine, Obihiro University (Japan). RAW264.7 macrophages are used to establish an *in vitro* model of *T. gondii* infection, and Vero cells are used to cultivate *T. gondii*. Both cells were cultured with DMEM high glucose medium plus 5% heat-inactivated FBS, 1% penicillin-streptomycin under 37°C, and 5% CO_2_ conditions. *T. gondii* was inoculated into precultured Vero cells and continued to culture. When some tachyzoites escaped from Vero cells, cells and tachyzoites were scraped and placed in a sterile culture dish, and then the cells were broken with a 5-micron needle filter and filtered to obtain tachyzoites.

### 2.4. Detection of RAW264.7 Macrophage Viability by MTT Method

RAW264.7 macrophage viability was detected by the MTT method. RAW264.7 macrophages cultured under standard conditions were adjusted to 4 × 10^5^ cells/mL and inoculated in a 96-well plate. Macrophages were treated with 0–400 *μ*g/mL concentrations of IOP diluted with DMEM for 24 h under 5% CO_2_ and 37°C. 20 *μ*L of MTT solution at 5 mg/mL concentration was added to each well and the cells were continually cultured for 4 h. 100 *μ*L DMSO was added to each well and shook at a low speed for 10 min. The absorbance was detected at 570 nm by a microplate reader. The cell survival rate (%) is calculated as follows: cell survival rate (%) = (OD value of test group − OD value of zero setting group)/(OD value of blank group − OD value of zero setting group) × 100%.

### 2.5. Detection of Cytokine Contents by ELISA

RAW264.7 macrophages cultured under standard conditions were adjusted to 5 × 10^5^ cells/mL and inoculated in a 24-well plate. The cells were infected with 2.5 × 10^6^/mL of *T. gondii* tachyzoites for 4 h (except for the normal group) and treated with different concentrations of IOP (100, 50, and 25 *μ*g/mL) for 12 h, the positive group was treated with SD (10 *μ*g/mL), the normal group was added with the same volume of culture medium, and the model group was only infected with tachyzoites. The supernatant of the cells in each well was collected, and the contents of cytokine IFN-*γ*, TNF-*α*, IL-1*β*, IL-4, and IL-6 in the supernatant were detected using ELISA kits. Briefly, mouse capture antibody was coated in a microwell plate and incubated at 4°C overnight and blocked with 5% BSA blocking solution at room temperature for 1 h. Samples from the supernatant of the cells were added separately and incubated for 2 h, followed by detection antibody for 1 h and avidin-HRP conjugate for 30 min. TMB chromogenic solution was added and incubated in the dark for 30 min, the reaction was stopped by adding 1 MH_2_SO_4_, and the OD value was measured at 450 nm on a microplate reader. The contents of cytokines were expressed based on the appropriate standard curve.

### 2.6. Detection of Cytokine and Chemokine mRNA Expression by RT-PCR

RAW264.7 macrophages cultured under standard conditions were adjusted to 4 × 10^6^ cells/mL and inoculated in a 6-well plate. The cells were infected with 2 × 10^7^/mL of *T. gondii* tachyzoites for 4 h (except for the normal group), and drug treatment was the same as above. The cell pellet in each group was collected, the total RNA of the cells was extracted, and 1 *μ*g of total RNA in each group was performed for RT-PCR. The PCR product was subjected to 1.5% agarose gel electrophoresis and developed with an E-Gel gel imaging system. The mRNA expression of cytokines and chemokines was analyzed with Quantity One Software. The primer sequences (Table 1) were designed by Primer Premier 5 (Premier, Canada) and synthesized by Invitrogen (Shanghai, China). *β*-Actin was used as an internal control.

### 2.7. Detection of Key Protein Expression in TLRs-NF-Κb/MAPKs Signaling Pathways by Western Blot

RAW264.7 macrophages cultured under standard conditions were adjusted to 3 × 10^6^ cells/mL and inoculated in a 25 cm^2^ culture flask. The cells were infected with 1.5 × 10^7^/mL of *T. gondii* tachyzoites for 4 h (except for the normal group), and drug treatment was the same as above. The cells in each group were washed with PBS and collected by centrifugation. The protein of the cells was extracted using RIPA buffer containing PMSF and separated by centrifugation, and the supernatant was aspirated. The concentration of protein was detected using the BCA method. Briefly, the standard curve was established using a serial of dilution of protein standard, the samples were placed in a 96-well plate, BCA solution was added and incubated at 37°C for 30 min, absorbance was measured at 562 nm on a microplate reader, and the concentration of protein was calculated based on the standard curve. The same amount of protein was taken for SDS-PAGE electrophoresis. The target protein was transferred into the PVDF membrane and the PVDF membrane was sealed in 5% skimmed milk for 2 h at room temperature and then incubated with primary antibodies p-IKK*α*/*β*, p-I*κ*B*α*, p-p65, p-p38, p38, p-ERK1/2, ERK1/2, p-JNK, JNK, TLR2, and TLR4 for overnight at 4°C, respectively. The primary antibody was washed with Tris-buffered saline/Tween 20 (TBST), and the membrane was incubated with secondary antibody for 1 h at room temperature. The membrane was washed with TBST; the target protein bands were developed in the WB imaging system with ECL color mixing solution. The gray values of the target protein in each group were analyzed by Quantity One Software.

### 2.8. Observation of p65 and c-Jun Activation by Immunocytochemistry

RAW264.7 macrophages cultured under standard conditions were adjusted to 5 × 10^5^ cells/mL and inoculated in a 35 mm glass-bottom dish. The cells were infected with 2.5 × 10^6^/mL of *T. gondii* tachyzoites for 4 h (except for the normal group), and drug treatment was the same as above. The supernatant of the cells in each group was sucked away and the cells cultured on a glass dish were gently washed with PBS. The cells were fixed with 4% paraformaldehyde for 30 min and 0.1% Triton X-100 was added for 15 min at room temperature to increase the permeability of membrane. The cells were saturated with PBS containing 5% BSA for 30 min at room temperature and processed for immunofluorescent staining with primary antibodies (p65, c-Jun) for 1 h, followed by Cy3-conjugated fluorescent secondary antibody for 1 h at room temperature. The cells were stained with DAPI in the dark for 15 min, and fluorescent signals of p65 and c-Jun activation and translocation were observed by fluorescent microscopy.

### 2.9. Statistical Analysis

All values are represented as means ± SD of results from at least three independent experiments. The differences between the groups were analyzed by one-way analysis of variance (ANOVA) and Student's *t*-test. All statistical analyses were performed by SPSS 20.0 statistical analysis software (SPSS, Inc., Chicago, IL, USA). *P*-value <0.05 was considered to be statistically significant.

## 3. Results

### 3.1. Chemical Characterization of IOP

**Figure 1:**
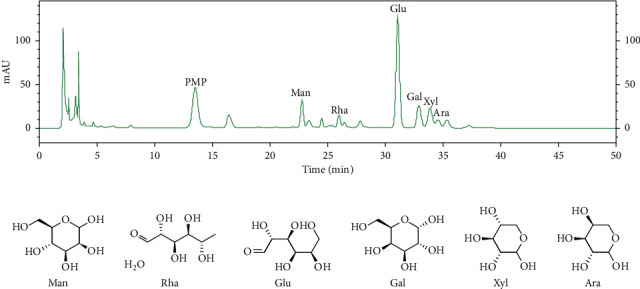
Chemical characterization of IOP. (a) HPLC profile of IOP. (b) Chemical structures of monosaccharides from IOP.

1-Phenyl-3-methyl-5-pyrazolone (PMP) was used as a derivatization reagent; the main chemical components from IOP were analyzed by the HPLC method. The monosaccharide compositions included Mannose (Man), Rhamnose (Rha), Glucose (Glu), Galactose (Gal), Xylose (Xyl), and Arabinose (Ara) with molar ratios of 2.2 : 1.1 : 11.8 : 2.8 : 2.7 : 1.0. The monosaccharide compositions and the chemical structures of monosaccharides from IOP are shown in Figure 1.

### 3.2. Effect of IOP on Cytokine Contents in RAW264.7 Macrophages Infected by *T. gondii*

**Figure 2:**
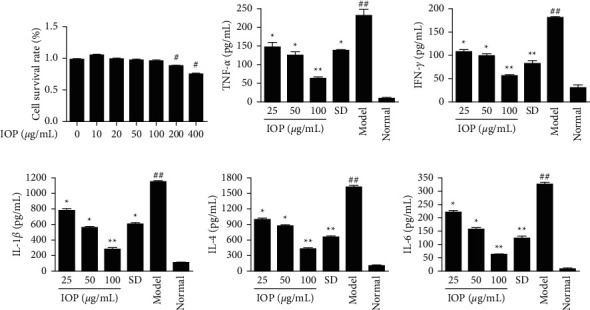
Effects of IOP on RAW264.7 cell viability and inflammatory cytokine contents in RAW264.7 macrophages infected with *T. gondii*. (a) RAW264.7 macrophages were treated with 0–400 *μ*g/mL of IOP for 12 h. The cell viability was measured by the CCK-8 method. (b–f) RAW264.7 macrophages were infected with *T. gondii* and treated with IOP or SD; the contents of inflammatory cytokines were determined using ELISA assay. Values are expressed as means ± SD of three independent experiments. ^#^*P* < 0.05, ^##^*P* < 0.01 vs. normal group; ^*∗*^*P* < 0.05, ^*∗∗*^*P* < 0.01 vs. model group.

The MTT method was used to detect the cytotoxicity of IOP on RAW264.7 cells to determine the safe doses of IOP. As shown in Figure 2(a), IOP at concentrations from 0 to 100 *μ*g/mL had no significant effect on the viability of RAW264.7 macrophages. Thus, the concentrations of IOP used in the following experiments were 25, 50, and 100 *μ*g/mL as the low, medium, and high dose groups of IOP, respectively.

The contents of cytokine TNF-*α*, IFN-*γ*, IL-1*β*, IL-4, and IL-6 in the supernatant of cells were detected using the ELISA method. As shown in Figures 2(b)–2(f), the contents of inflammatory cytokines in the model group were increased compared with those in the normal group (*P* < 0.01). However, their contents in 25, 50, and 100 *μ*g/mL IOP groups were decreased compared with the model group (*P* < 0.05 or *P* < 0.01). It indicated that IOP could inhibit the excessive secretion of inflammatory cytokines caused by *T. gondii* infection from RAW264.7 macrophages. SD also inhibited the secretion of these inflammatory cytokines (*P* < 0.05 or *P* < 0.01).

### 3.3. Effect of IOP on Cytokine and Chemokine mRNA Expression in RAW264.7 Macrophages Infected by *T. gondii*

**Figure 3:**
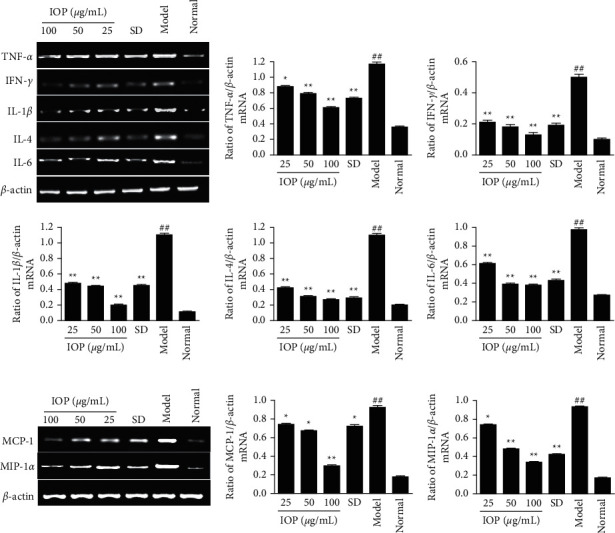
Effects of IOP on the mRNA expression of inflammatory cytokines (a) and chemokines (b) in RAW264.7 macrophages infected with *T. gondii*. RAW264.7 macrophages were infected with *T. gondii* and treated with IOP or SD; the mRNA expression of inflammatory cytokine TNF-*α*, IFN-*γ*, IL-1*β*, IL-4, IL-6, and chemokine MIP-1, MCP-1 was determined by RT-PCR assay. Values are expressed as means ± SD of three independent experiments. ^##^*P* < 0.01 vs. normal group; ^*∗*^*P* < 0.05, ^*∗∗*^*P* < 0.01 vs. model group.

The cytokine and chemokine mRNA expression was detected by RT-PCR and quantitatively analyzed. As shown in Figure 3(a), the mRNA expression levels of cytokines TNF-*α*, IFN-*γ*, IL-1*β*, IL-4, and IL-6 in the model group were increased compared with those in the normal group (*P* < 0.01). However, the mRNA expression levels of these cytokines in 25, 50, and 100 *μ*g/mL IOP groups were decreased compared with the model group (*P* < 0.05 or *P* < 0.01). As shown in Figure 3(b), the mRNA expression levels of chemokines MCP-1 and MIP-1*α* in the model group were increased compared with those in the normal group (*P* < 0.01). However, the mRNA expression levels of chemokines MCP-1 and MIP-1*α* in 25, 50, and 100 *μ*g/mL IOP groups were decreased compared with those in the model group (*P* < 0.05 or *P* < 0.01). SD also decreased the mRNA expression levels of these cytokines and chemokines (*P* < 0.05 or *P* < 0.01).

### 3.4. Regulation of IOP on NF-Κb and MAPKs Signaling Pathways in RAW264.7 Macrophages Infected by *T. gondii*

**Figure 4:**
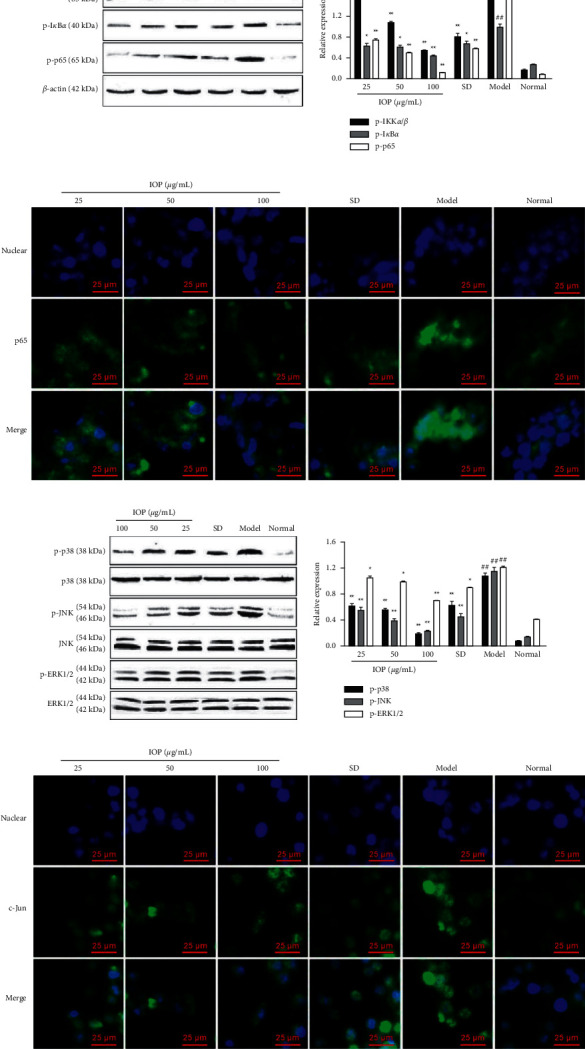
Regulation of IOP on NF-*κ*B (a) and (b) and MAPKs (c) and (d) signaling pathways in RAW264.7 macrophages infected with *T. gondii*. (a, c) RAW264.7 macrophages were infected with *T. gondii* and treated with IOP or SD; the phosphorylated levels of IKK*α*/*β*, I*κ*B*α*, NF-*κ*B p65, p38, JNK, and ERK1/2 were determined by Western blot analysis. Values are expressed as means ± SD of three independent experiments. ^##^*P* < 0.01 vs. normal group; ^*∗*^*P* < 0.05, ^*∗∗*^*P* < 0.01 vs. model group. (b, d) RAW264.7 macrophages were infected with *T. gondii* and treated with IOP or SD; NF-*κ*B p65 and c-Jun activation was observed by immunocytochemistry staining. Blue represents nucleus; green represents NF-*κ*B p65 and c-Jun. The image magnification is 400×.

The phosphorylated levels of key proteins in NF-*κ*B and MAPKs signaling pathways were detected by Western blot. As shown in Figures 4(a) and 4(c), the levels of p-IKK*α*/*β*, p-I*κ*B*α*, p-p65 of NF-*κ*B signaling pathway and p-p38, p-JNK, and p-ERK1/2 of MAPKs signaling pathway in model group were increased compared with those in the normal group (*P* < 0.01), while the expressions of these proteins in 25, 50, and 100 *μ*g/mL IOP groups and SD group were decreased compared with the model group (*P* < 0.05 or *P* < 0.01). These evidenced that IOP and SD could inhibit key protein IKK*α*/*β*, I*κ*B*α* and p65 phosphorylation of NF-*κ*B signaling pathway and p38, and JNK and ERK1/2 phosphorylation of MAPKs signaling pathway in *T. gondii*-infected RAW264.7 macrophages.

### 3.5. Effect of IOP on NF-Κb p65 and c-Jun Activation in RAW264.7 Macrophages Infected by *T. gondii*

The effect of IOP on p65 and c-Jun activation was further evaluated by immunocytochemistry staining in *T. gondii*-infected RAW264.7 macrophages. As shown in Figures 4(b) and 4(d), p65 and c-Jun were mainly distributed in the cytoplasmic compartment in normal groups, c-Jun and p65 were activated, and most of them were translocated from cytoplasm to nucleus in model groups. However, the activation and nuclear translocation of p65 and c-Jun induced by *T. gondii* infection could be inhibited in 25, 50, and 100 *μ*g/mL IOP groups and SD group.

### 3.6. Effect of IOP on TLR2 and TLR4 Protein Expression in RAW264.7 Macrophages Infected by *T. gondii*

**Figure 5:**
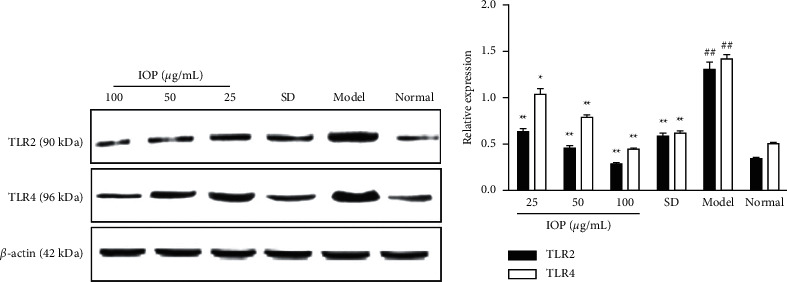
Effects of IOP on TLR2 and TLR4 protein expression in RAW264.7 macrophages infected with *T. gondii*. RAW264.7 macrophages were infected with *T. gondii* and treated with IOP or SD; the protein expression of TLR2 and TLR4 was determined by Western blot analysis. Values are expressed as means ± SD of three independent experiments. ^##^*P* < 0.01 vs. normal group; ^*∗*^*P* < 0.05, ^*∗∗*^*P* < 0.01 vs. model group.

The protein expression of TLR2 and TLR4 was determined by Western blot. As shown in Figure 5, the expression levels of TLR2 and TLR4 in the model group were upregulated compared with those in the normal group (*P* < 0.01). However, the levels of TLR2 in 25, 50, and 100 *μ*g/mL IOP groups were downregulated compared with those in the model group (*P* < 0.01); the expression levels of TLR4 in 25, 50 and 100 *μ*g/mL IOP groups and SD group were downregulated compared with those in the model group (*P* < 0.05 or *P* < 0.01). These showed that IOP and SD could downregulate the overexpression of TLR2 and TLR4 in RAW264.7 macrophages infected with *T. gondii*.

## 4. Discussion

Toxoplasmosis is characterized by excessive or imbalanced inflammation and pathology [23]. *T. gondii* invades the organisms and converts them to the rapidly dividing tachyzoite form, inducing a series of inflammatory responses, which causes pathological damage to invasive tissues and cells [24]. Host cell invasion is essential for the pathogenicity of the obligate intracellular protozoan parasite *T. gondii*. As important immune cells, macrophages play an important role in directing the host's immune response to infection and also play a pathogenic role in inflammatory disorders by producing excessive inflammatory mediators [25]. Therefore, in the present study, mouse RAW264.7 macrophages were used to establish the *in vitro* model of *T. gondii* infection and explore the anti-inflammatory effect and mechanism of IOP against *T. gondii* infection *in vitro*. Our data revealed that IOP inhibited the inflammatory response caused by *T. gondii* infection in RAW264.7 macrophages by suppressing inflammatory cytokines and chemokines. Further, we found these effects were mediated by inhibiting the TLR2/TLR4-NF-*κ*B/MAPKs signaling pathways.

*T. gondii* infection is recognized by immune sensors, leading to the production of cytokines and chemokines [26]. Cytokines are a class of proteins or small molecular peptides that can transmit information among cells; they are considered to be important initiators of the inflammatory responses and mediators of the development of infectious diseases [27]. TNF-*α* and IL-1*β* are critical inflammatory cytokines and play a significant role in regulating the host's immune defense against *T. gondii* infection [28]. Besides, IFN-*γ* and IL-6 are also important cytokines in the inflammatory response and show considerable value in the process of *T. gondii* infection [29, 30]. IL-4 is a multifunctional cytokine and possesses a two-way regulation function; it has an antagonistic effect on proinflammatory cytokines in the early stage of *T. gondii* acute infection, but the excessive or long-term accumulation of IL-4 will promote the massive reproduction of tachyzoites [31]. In this study, *T. gondii* infection led to excessive accumulation of IL-4 and promoted the massive reproduction of tachyzoites, which aggravated the inflammatory response. Therefore, as the essential indicator of immune regulation and inflammatory response, abnormal production of a particular cytokine would lead to a perturbation of homeostasis and may contribute to the pathogenesis of certain autoimmune or inflammatory diseases [32]. Our study showed that IOP could inhibit the excessive secretion and mRNA expression of cytokines IFN-*γ*, TNF-*α*, IL-1*β*, IL-4, and IL-6 in RAW264.7 macrophages infected by *T. gondii.* These results are consistent with our reported results *in vivo* [19, 20].

Chemokines are a subset of chemotactic polypeptides, which are critical mediators of both leukocyte activation and chemotaxis. Chemokines are released by a variety of immune cells in response to infection and attract cell migration to the site of infection. [33, 34] MCP-1 and MIP-1*α* are cysteine-cysteine (CC) chemokine family. They function as major chemoattractant factors for lymphocytes and monocytes and are involved in controlling *T. gondii* infection and pathogenesis [35]. It has been shown that the expressions of MIP-1*α* and MCP-1 gene transcripts are upregulated in *T. gondii*-stimulated macrophages and fibroblasts [35, 36]. Our present study also showed that *T. gondii* infection remarkably increased chemokine MIP-1*α* and MCP-1 mRNA expression, while IOP could suppress their expression levels in RAW264.7 macrophages infected by *T. gondii*.

TLRs exert a critical role in host defense against various microbial pathogens and are also key innate receptors for recognizing ligands expressed by *T. gondii* [37]. Among TLRs, TLR2 and TLR4 are involved in host defense against *T. gondii* infection through activation [10]. It has been reported that the increased levels of TLR2 and TLR4 may play an important role during acute *T. gondii* infection [38]. The activation of TLR2 and TLR4 triggers the downstream NF-*κ*B and MAPKs signaling pathways, which further promotes the synthesis and secretion of cytokines and chemokines by activating a variety of transcription factors [10–12]. NF-*κ*B is typically a heterodimer of RelA (p65) and NF-*κ*B1 (p50). In resting cells, NF-*κ*B is bound to the inhibitory protein I*κ*B and retained in an inactive form in the cytoplasm. When stimulated, I*κ*B is phosphorylated and degraded by I*κ*B kinase (IKK), and NF-*κ*B is released from I*κ*B and translocates into the nucleus to initiate gene expression. [39, 40] When *T. gondii* invades cells, extracellular signals are transmitted into cells through membrane receptors, which can activate downstream IKK, induce I*κ*B phosphorylation, and ultimately promote the nuclear translocation of NF-*κ*B subunits [41, 42]. Our present study found that key proteins IKK*α*/*β*, I*κ*B*α*, and p65 in NF-*κ*B signaling pathway were highly phosphorylated in *T. gondii-*infected RAW264.7 cells, while IOP could inhibit IKK*α*/*β*, I*κ*B*α*, and p65 phosphorylation and prevent the activation and translocation of p65 from the cytoplasm into the nucleus. These suggested that IOP inhibited the inflammatory response by blocking TLRs/NF-*κ*B signaling pathway in RAW264.7 macrophages infected with *T. gondii*.

As another important signal transduction pathway, the MAPKs pathway appears to be involved in the invasion process of various microorganisms. MAPKs family includes JNK, ERK1/2, and p38; the activation of JNK, ERK1/2, and p38 MAPKs occurs during the invasion and proliferation of *T. gondii* tachyzoites in HeLa cells. The increased secretion and expression of MIP-1*α* and MCP-1 are also detected in infected macrophages and appear to occur through the MAPKs signaling pathway [36]. MAPKs phosphorylation can be triggered by stimulation, resulting in the increased expression of p-MAPKs, including p-JNK, p-ERK, or p-p38 [43]. The ability of *T. gondii* to trigger specific phosphorylation of ERK1/2, JNK, and p38 MAPKs has been observed in monocytic cells [12]. JNK acts on the transcriptional activation domain of c-Jun, and phosphorylated c-Jun is transferred to the nucleus, where AP-1 activation is induced and participates in the inflammatory response [44]. Our present study found that ERK1/2, JNK, and p38 kinases were highly phosphorylated in *T. gondii-*infected RAW264.7 macrophages, while IOP could inhibit ERK1/2, JNK, and p38 phosphorylation and prevent the activation and translocation of transcription factor c-Jun from the cytoplasm to the nucleus. These suggested that IOP may inhibit the inflammatory response by blocking TLRs/MAPKs signaling pathway in RAW264.7 macrophages infected with *T. gondii*.

**Figure 6:**
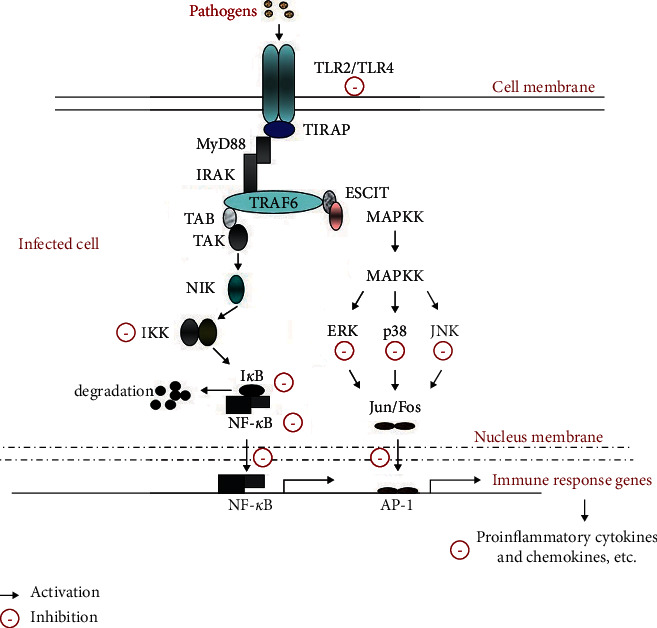
A schematic summary for possible immunomodulatory signaling mechanisms of IOP in *T. gondii*-infected RAW264.7 macrophages in the present study.

In conclusion, our results showed that IOP could inhibit the overexpression of inflammatory cytokines and chemokines in *T. gondii*-infected RAW264.7 macrophages. The inhibitory effects of IOP were attributed to the inhibition of TLR2/TLR4-NF-*κ*B/MAPKs signaling pathways (Figure 6). Taken together, the present study indicates that IOP inhibits the excessive inflammatory response caused by *T. gondii* infection and exerts the *in vitro* anti-*T. gondii* effect through the immunomodulatory mechanism. It provides the theoretical basis and evidence for preventing and treating *T. gondii* infection with IOP.

## Figures and Tables

**Table 1:** The primer sequences used for RT-PCR.

Genes	Forward primer (5′-3′)	Reverse primer (5′-3′)
IFN-*γ*	CGCTACACACTGCATCTTGG	TTCCACTCTATGCCACTTGAG
TNF-*α*	AGAATGAGGCTGGATAAGA	AGAGGTTCAGTGATGTAGCG
IL-1*β*	TTCAAGGGGACATTAGGCAG	TGTGCTGGTGCTTCATTCAT
IL-4	AACGAGGTCACAGGAGAAGG	TGGAAGCCCTACAGACAAGC
IL-6	GCCTTCTTGGGACTGATG	CTGGCTTTGTCTTTCTTGTT
MIP-1*α*	CCACTGCCCTTGCTGTTCTT	GGCATTCAGTTCCAGGTCAG
MCP-1	AGAGAGCCAGACGGGAGGAA	GTAGCAGCAGGTGAGTGGGG
*β*-Actin	CTGTCCCTGTATGCCTCTG	ATGTCACGCACGATTTCC

## Data Availability

The data that support the findings of this study are available from the corresponding author upon reasonable request.
